# Investigating the association between medication regimen complexity, medication adherence and treatment satisfaction among Malaysian older adult patients: a cross-sectional study

**DOI:** 10.1186/s12877-024-05016-y

**Published:** 2024-05-22

**Authors:** Mohammad Adam Al Haqimy Mohammad Yunus, Muhammad Eid Akkawi, Abdul Rahman Fata Nahas

**Affiliations:** 1 Department of Pharmacy Practice, Faculty of Pharmacy, International Islamic University Malaysia, Kuantan, Malaysia; 2 Quality Use of Medicines Research Group, Faculty of Pharmacy, International Islamic University Malaysia, Kuantan, Malaysia

**Keywords:** Older adults, Medication adherence, Treatment satisfaction, medication regimen complexity, Outpatients, Malaysia

## Abstract

**Background:**

The prevalence of medication nonadherence among Malaysian older adults is approximately 60%. However, there is a lack of studies assessing the factors associated with medication nonadherence among this population. This research aims to explore the association between medication regimen complexity (MRC), treatment satisfaction and medication adherence among Malaysian older adults.

**Method:**

A cross-sectional study was conducted in outpatient clinics of a teaching hospital in Pahang, Malaysia, between April 2023 and September 2023. MRC Index (MRCI), Treatment Satisfaction for Medication version II (TSQM v.II), and the Malaysian Medication Adherence Assessment Tool (MyMAAT) were used. Multivariate linear and logistic regression models were performed to test the factors affecting treatment satisfaction and medication adherence. Mediator analysis was implemented to assess the mediating role of treatment satisfaction.

**Result:**

The study involved 429 Malaysian older adult patients, with a prevalence of nonadherence of 51.0% (*n* = 219) and an MRCI mean score of 17.37 (SD = 7.07). The mean overall treatment satisfaction score was 73.91 (SD = 15.23). Multivariate logistic regression analysis expressed four significant predictors associated with nonadherence: MRC (AOR = 1.179, *p* = 0.002), overall treatment satisfaction (AOR = 0.847, *p* < 0.001), partially self-managed medication (AOR = 2.675, *p* = 0.011) and fully managed medication by family members/caregivers (AOR = 8.436, *p* = 0.004). Multivariate linear regression shows three predictors of treatment satisfaction: MRC (β = -1.395, *p* < 0.001), Charlson Comorbidity Index (CCI) (β = -0.746, *p =* 0.009) and self-managed medication (β = 5.554, *p* = 0.006). Mediator analysis indicated that treatment satisfaction partially mediated the association between MRC and nonadherence.

**Conclusion:**

Nonadherence was quite prevalent among Malaysian older outpatients and was associated with regimen complexity, treatment satisfaction and patient dependence on others to manage their medications. Future studies should focus on interventions to control the factors that negatively affect patients’ medication adherence.

## Introduction

Malaysia has changed its status to be an aging nation, as the population of 65 years and above has already reached approximately 7.3% in the last quarter of 2022 [1]. In addition, the declaration of this status attainment in Malaysia is earlier than expected, which was forecasted to be in 2030. The significant growth of the older adult population is mainly because of the increase in average life expectancy and the decrease in the mortality rate [2]. Therefore, the anticipated expenditure by the government will increase to cover the healthcare sector, as older adult patients are usually diagnosed with multiple comorbidities and in greater need of healthcare services [2].

Older adults usually have multiple comorbidities that require the use of multiple medications to control these conditions. However, medication adherence among this population is still suboptimal [3]. A meta-analysis reported that the prevalence of nonadherence to medication among older adult patients in Malaysia is approximately 60% [4]. Poor medication adherence is believed to be the critical reason for treatment failure, where the treatment and clinical outcomes are not achieved [5]. Poor medication adherence is a major challenge experienced by the older adult population, and it is highly associated with unfavorable clinical outcomes of their medical conditions [6]. Nonadherent older adult patients are prone to receive more medications and even overtreatment [7]. The average cost of returned and unused medication in the outpatient pharmacy department in a Malaysian hospital was high, approximately 100 Ringgit Malaysia (RM)/USD 22$ per patient [8]. Based on that, the estimated cost of nonadherence exceeds a few million RM per annum. This means that the nonadherence issue causes a substantial financial burden to the healthcare system in the long run. Therefore, all stakeholders should focus intensely on the problems regarding nonadherence among older adult patients.

**Fig. 1:**
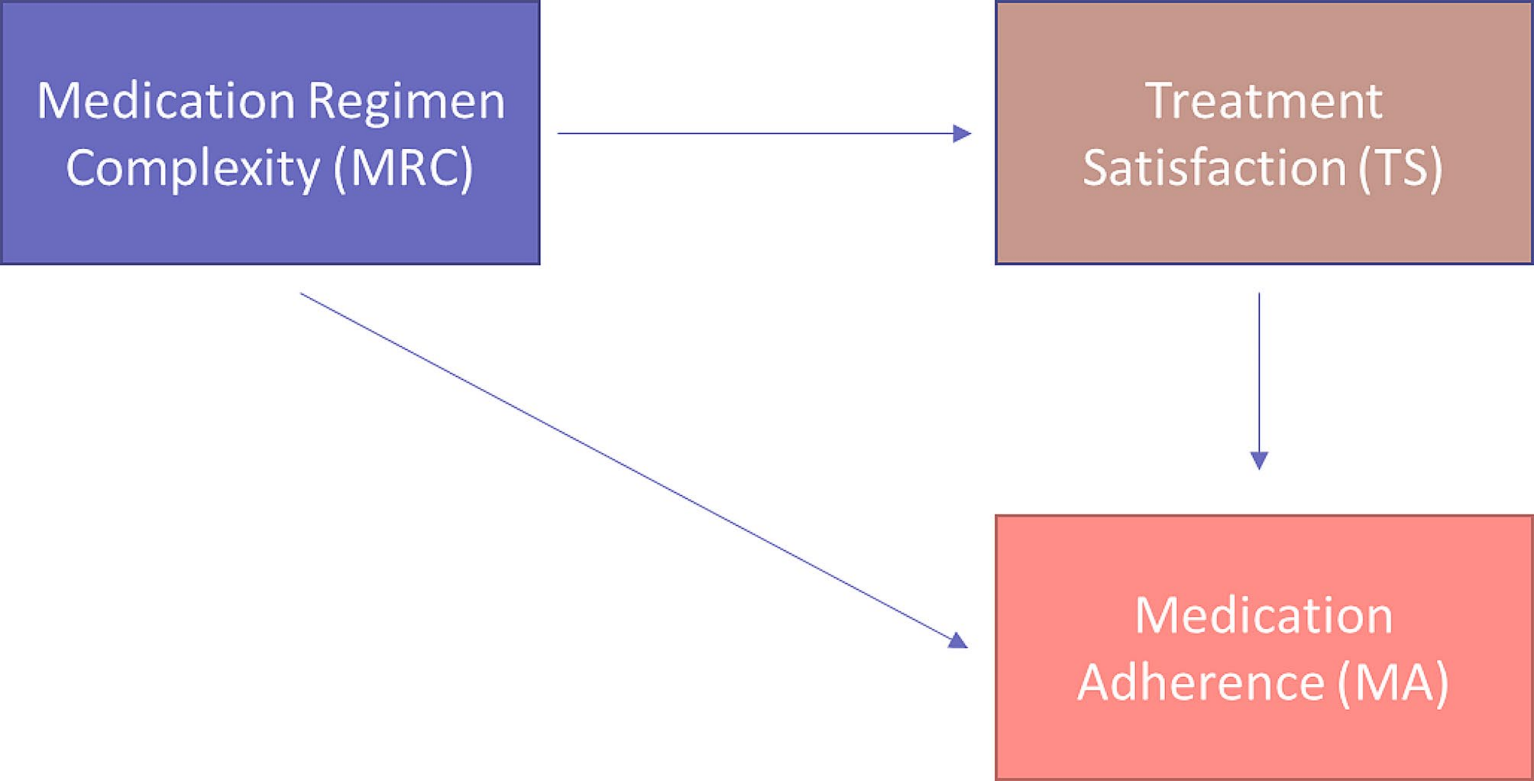
Framework of the association between medication regimen complexity, treatment satisfaction and medication adherence. MRC as an independent variable, TS as a mediator variable and MA as a dependent variable

Factors associated with nonadherence among the older adult population can be identified as patient-related, medication-related, physician-related, system-based and other factors. Precisely, medication-related factors include polypharmacy, medication regimen complexity and modification in the regimen [9]. Patient-related factors encompass demographic characteristics, treatment satisfaction, patient behaviors and dependency in medication management [9] The role of the caregiver in managing the patient’s medication is a vital component for addressing nonadherence issues among older adult patients [9]. Also, it was proposed that treatment satisfaction and medication regimen complexity (MRC) are key determinants of nonadherence. Wiffen et al. proposed a similar finding: treatment satisfaction and MRC are the determinants of nonadherence [10]. Thus, our research addresses the proposed relationship between these three components, as shown in Fig. 1.

Treatment satisfaction is interpreted as a patient-reported outcome measure (PROM), which is the total of the patient’s satisfaction with the perception and expectation of the treatment given [11]. It has been demonstrated that treatment satisfaction is determined by the satisfaction with medication’s effectiveness, the convenience of medication administration, and the presence of undesirable side effects [11]. The development of a generic measurement tool enabled researchers to evaluate the level of treatment satisfaction in different patients. A commonly used tool is the Treatment Satisfaction Questionnaire for Medication (TSQM) [12].

Previously, it was known that MRC could be explained as a simple count of medications administered per patient. However, the number of medications taken cannot quantify the complexity measure of the medication regimen, which also must describe the various routes of administration, dosage forms, dosing frequencies and additional medication instructions [13]. A study by George et al. (2004) quantified the medication regimen complexity, known as the Medication Regimen Complexity Index (MRCI) [14].

To the best of our knowledge, there is a scarcity of conclusive findings regarding the studies investigating the association between medication regimen complexity, treatment satisfaction, and medication adherence altogether, especially among Malaysian older adults. Thus, the objectives of this research were: to study the impact of medication regimen complexity and other variables on medication adherence and treatment satisfaction, to determine the association between treatment satisfaction and other variables with medication adherence, and to identify the mediator variable in the proposed framework using mediator analysis.

## Methodology

### Study design and settings

A cross-sectional study was conducted among older adult patients visiting outpatient clinics at Sultan Ahmad Shah Medical Centre at the International Islamic University Malaysia (SASMEC@IIUM), which is a teaching hospital in Pahang, Malaysia. The study was conducted between April 2023 and September 2023 in various outpatient clinics under the department of orthopedic, traumatology and rehabilitation unit as well as the department of internal medicine. Convenience sampling technique was used in this research.

### Sample size

The sample size was calculated using the Raosoft sample size calculator. For this research, the margin of error was set at 5%, the confidence level was 95%, the population size was 20,000, and the prevalence of nonadherence was 60% [4]. The exact population size of older adults cannot be determined in SASMEC@IIUM. Therefore, the selection of 20,000 is the safest option, since the sample size calculated would not change very much once the population exceeds that number. Thus, the recommended sample size for this study was approximately 363 patients. An additional 20% patients were included in the sample size, accounting for the contingency of possible attrition and nonresponse of participants in the study to achieve enough statistical power.

### Study population

Participants were recruited on the day of their routine appointment and consultation with their respective clinics, where the patient was invited to participate in the study. The general aim of the study and ethical considerations were explained briefly. The selection criteria for the participants in this study were as follows:

1. Malaysian citizens.
2. Older adult outpatients aged 65 years old and above;
3. Patients who had been using at least three prescribed chronic medications for the past three months;
4. Consented to take part in the study;
5. Able to speak and understand the Malay language.

The patients were excluded from the study if they were unable to give proper informed consent (e.g., patients with cognitive impairment such as dementia or Alzheimer’s disease) or enrolled in another clinical study. Patients who had missing information in their electronic health records were also excluded.

### Data collection

Data were collected by in-person interviews with the patients and reviewing their paper and electrical medical records. The collected data comprised sociodemographic information including age, gender, ethnicity, marital status, education level, participant’s monthly income, employment status, geographical location, employment status, and medical information such as the current diseases and chronic medications.

### Operational definitions and measurement tools

Polypharmacy and hyperpolypharmacy were defined as concurrent use of ≥ 5 and ≥ 10 medications, respectively [15].

The Charlson Comorbidity Index (CCI) was used to evaluate the clinimetric properties of the patient’s morbidity [16]. A CCI score of 1–2 represents mild comorbidities, whereas 3–4 and ≥ 5 represents moderate and severe comorbidities, respectively [17].

Diseases were classified using the International Classification of Diseases 11^th^ revision (ICD-11), which is a global standard for reporting the classification of health and diagnosis information [18].

The medications were classified based on the Anatomical Therapeutic Chemical (ATC) classification, which considers the active ingredient of the drug in proportion to body systems [19].

#### Medication regimen complexity (MRC)

MRC was measured using the medication regimen complexity index (MRCI), a validated 65-item instrument that measures and quantifies the complexity of the patient’s overall medication regimen [14]. There are three components in this measurement tool: dosage form, dosing frequency and additional instructions. The MRCI score was divided into three categories: low complexity with a score < 15, moderate complexity with a score ranging from 15.5 to 20, and high complexity with a score above 20.5 [20]. Additionally, we used automated calculation of MRCI by using Microsoft Access v1.0 to ease the calculation [21].

#### Medication adherence

The Malaysia Medication Assessment Adherence Tool (MyMAAT) which is a self-reported measurement was used to assess the patient’s adherence to the medication. It is a validated tool that is readily available in Malay and English languages. This 12-item questionnaire has a 5-point Likert-type scale ranging from 1 “Strongly Agree” to 5 “Strongly Disagree”. The minimum total score is 12 points, and the maximum is 60 points. The cutoff point suggested by the developers is 54. A score ≥ 54 indicates good adherence, whereas a total score below 54 indicates moderate and poor adherence [22].

#### Treatment satisfaction

Treatment Satisfaction Questionnaire for Medication (TSQM v. II) was used to measure patients’ satisfaction with their treatment. This 11-item questionnaire is divided into four domains: effectiveness, side effects, convenience, and general satisfaction. Five- and seven-point Likert-type scales were used to evaluate individuals’ responses [23]. The questionnaire was used after obtaining permission from the copyright owners (IQVIA group, you may refer to www.iqvia.com/tsqm).

In this measurement tool, effectiveness (items 1–2), convenience (items 7–9), and general satisfaction (items 10–11) domains have 7-point Likert-type scales ranging from 1 “Extremely Dissatisfied” to 7 “Extremely Satisfied”. The side effects domain (items 3–6) had one dichotomous scale (presence of side effects) of “Yes” and “No” and three items of 5-point Likert-type scales ranging from 1 “Extremely Dissatisfied” to 5 “Not at all Dissatisfied”. The score was calculated as per the algorithm provided by the developers. Each TSQM domain score ranges from 0 to 100, with higher values indicating more patient satisfaction.

#### Medication management

Medication management was assessed by asking the patients whether they managed their medications by themselves or by some assistance from caregivers or totally by caregivers. Based on that, medication management was categorized as self-managed by the patient, partially self-managed, or fully managed by family members/caregivers. In fact, medication management was assessed by one straightforward question because we believe it is sufficient to represent the overall status of medication management as a variable included in the applied regression models. We have not used a comprehensive tool to assess this aspect of medication management for two reasons. First, there is no such generic validated tool available in Malay language. Second, we wanted to avoid overburdening the participants by answering too many questionnaires which could affect the credibility of their answers due to weariness.

### Statistical analysis

#### Descriptive analysis

All statistical analyses were performed using SPSS v.27.0. For descriptive data, continuous variables were analyzed as the mean and standard deviation (SD), while categorical variables were analyzed as frequency and percentage. All data were sorted and screened for any extreme cases/outliers and normality of the data. The normality test evaluated data using skewness and kurtosis [24, 25]. Mahalanobis distance was the analysis used to detect extreme cases/outliers [26].

#### Test of relationship

Factors that might be associated with either treatment satisfaction or medication adherence were tested using univariate and multivariate analyses. Chi-square test, *t* test and simple linear regression were used for univariate analyses. Binomial logistic regression was applied to test the predictors of medication nonadherence, while multiple linear regression was used to check factors associated with treatment satisfaction and MRC. Logistic regression was computed because medication nonadherence is binary variable, while linear regression was computed because treatment satisfaction and MRC were treated as continuous variables. Hosmer‒Lemeshow’s test and area under the receiver operating curve (ROC) were used to determine the goodness of fit of the regression models. We also used the mediation analysis proposed by Hayes to explore the direct and indirect effect of medication regimen complexity on medication adherence with treatment satisfaction as a mediator variable [27]. Figure 1.

## Result

### Sociodemographic and clinical characteristics of the sample

The mean age of the participants was 71.98 (SD = 5.48) years old. The proportion of male patients (57.6%, *n* = 247) was slightly higher than that of female patients. The number of patients from urban areas was 246, which represents 57.3% of the study sample. Table 1. For clinical characteristic data, the mean score for the CCI was 5.35 (SD = 2.08), along with approximately more than half of the sample categorized as having severe CCI (60.1%, *n* = 258). Regarding outcomes, the prevalence of nonadherence was 51.0% (*n* = 219). Meanwhile, the mean values for the treatment satisfaction score for each domain were 70.69 (SD = 19.38) for effectiveness, 87.37 (SD = 21.78) for side effects, 65.59 (SD = 21.18) for convenience and 71.99 (SD = 18.85) for general satisfaction. Last, the mean score for the MRCI was 17.37 (SD = 7.07), with moderate to high complexity accounting for 53.3% (*n* = 230) of the total participants involved in this research. Table 1.

**Table 1:** Demographic and medical characteristics of the patients (*n* = 429)

Variable	Overall patients (%)	Adherent (*n* = 210)	Non-Adherent (*n* = 219)	*p* value
Age (mean ± SD)	71.98 ± 5.48	71.50 ± 5.07	72.44 ± 5.83	0.073
65–69 years old	157 (36.6)	79 (18.4)	78 (18.2)	0.052
70–74 years old	152 (35.4)	83 (19.3)	69 (16.1)	
≥ 75 years old	120 (28.0)	48 (11.2)	72 (16.8)	
Gender				
Male	247 (57.6)	113 (26.3%)	134 (31.2%)	0.122
Female	182 (42.4)	97 (22.6%)	85 (19.8%)	
Ethnicity				
Malay	354 (82.5)	168 (39.2)	186 (43.3)	0.179
Non - Malay	75 (17.5)	42 (9.8)	33 (7.7)	
Marital Status				
Married	338 (78.8)	173 (40.3)	165 (38.5)	0.075
Others (widowed, divorced or single)	91 (21.2)	37 (8.6)	54 (12.6)	
Residency				
Urban	246 (57.3)	118 (27.5)	128 (29.8)	0.637
Rural	183 (42.7)	92 (21.4)	91 (21.2)	
Education Level				
College/University	181 (42.2)	90 (21.0)	91 (21.2)	0.234
Secondary school	152 (35.4)	80 (18.6)	72 (16.8)	
Primary school or no formal education	96 (22.4)	40 (9.3)	56 (13.1)	
Employment Status				
Pensioner	219 (51.0)	110 (25.6)	109 (25.4)	0.246
Unemployed	91 (21.2)	38 (8.9)	53 (12.4)	
Housewife	92 (21.4)	45 (10.5)	47 (11.0)	
Self-employed & employed	27 (6.3)	17 (4.0)	10 (2.3)	
Income per month				
< RM 1000	148 (34.5)	66 (15.4)	82 (19.1)	0.484
RM 1000 - RM 2500	93 (21.7)	52 (12.1)	41 (9.6)	
RM 2501 - RM 4000	66 (15.4)	30 (7.0)	36 (8.4)	
RM 4001 - RM 4999	54(12.6)	28 (6.5)	26 (6.1)	
≥ RM 5000	68(15.9)	34 (7.9)	34 (7.9)	
Management of Medications				
Self-managed	217 (50.6)	162 (37.8)	55 (12.8)	< 0.001^a^
Partially self-managed	168 (39.2)	42 (9.8)	126 (29.4)	
Fully managed by family members/caregivers	44 (10.3)	6 (1.4)	38 (8.9)	
Charlson Comorbidity Index				
CCI (mean ± SD)	5.35 ± 2.08	4.78 ± 1.89	5.90 ± 2.10	< 0.001^b^
Mild CCI (≤ 2)	21 (4.9)	17 (4.0)	4 (0.9)	< 0.001^a^
Moderate CCI (3-4)	150 (35.0)	91 (21.2)	59 (13.8)	
Severe CCI (≥ 5)	258 (60.1)	102 (23.8)	156 (36.4)	
Treatment Satisfaction (mean ± SD)				
Effectiveness	70.69 ± 19.38	82.14 ± 13.04	59.70 ± 18.08	< 0.001^b^
Side Effects	87.37 ± 21.78	93.06 ± 15.57	81.93 ± 25.23	< 0.001^b^
Convenience	65.59 ± 21.18	79.23 ± 14.60	52.51 ± 18.02	< 0.001^b^
General Satisfaction	71.99 ± 18.85	86.03 ± 11.03	58.52 ± 14.44	< 0.001^b^
Overall Mean Treatment Satisfaction	73.91 ± 15.23	85.12 ± 8.67	63.17 ± 12.10	< 0.001^b^
Medication Regimen Complexity				
Total MRCI (mean ± SD)	17.38 ± 7.07	12.76 ± 4.27	21.81 ± 6.35	< 0.001^b^
Component A (Dosage Form) (mean ± SD)	3.62 ± 2.77	2.20 ± 1.77	4.99 ± 2.88	< 0.001^b^
Component B (Dosing Frequency) (mean ± SD)	10.09 ± 4.31	7.80 ± 2.69	12.29 ± 4.43	< 0.001^b^
Component C (Additional Instruction) (mean ± SD)	3.67 ± 1.88	2.77 ± 1.50	4.53 ± 1.81	< 0.001^b^
Low complexity (≤ 15)	199 (46.4)	169 (39.4)	30 (7.0)	< 0.001^a^
Medium complexity (15.5–20)	100 (23.3)	32 (7.5)	68 (15.9)	
High complexity (≥ 20.5)	130 (30.3)	9 (2.1)	121 (28.2)	
Prescribed Medications				
Per Patient (mean ± SD)	7.69 ± 2.59	6.37 ± 1.96	8.95 ± 2.49	< 0.001^b^
No polypharmacy (3–4 medications)	42 (9.8)	35 (8.2)	7 (1.6)	< 0.001^a^
Polypharmacy (5–9 medications)	288 (67.1)	166 (38.7)	122 (28.4)	
Hyperpolypharmacy (≥ 10 medications)	99 (23.1)	9 (2.1)	90 (21.0)	

Note: ^a^ Statistically significant *p* values from continuous variables using *t* test; ^b^ Statistically significant *p* values from categorical variables using χ^2^ test; Based on normality test, the normally distributed data was reported as mean and standard deviation values (mean ± SD), while the non-normally distributed data was expressed as median values.

Based on the ICD-11 classification, the most common comorbidities were circulatory system diseases (44.8%), followed by endocrine, nutritional and metabolic diseases (15.5%) and genitourinary system diseases (9.9%). An average of 7.69 (SD = 2.59) prescribed medications per participant was found, with 90.2% (*n* = 387) of the participants having polypharmacy. Based on ATC classification, common types of prescribed chronic medication were those pertaining to cardiovascular system (37.0%, *n* = 1227), alimentary tract & metabolism (27.1%, *n* = 898) and blood & blood-forming organs (11.5%, *n* = 380) (Table 2). In addition, approximately half of the participants were able to manage their medications independently (50.6%, *n* = 217).

**Table 2:** Diseases and prescribed medications of the study patients (*n* = 429)

Variable	Frequency
Therapeutic groups according to ATC classification	**Number of medication (%)** (***n*** = **3315)**
Cardiovascular system	1227 (37.0)
Alimentary tract & metabolism	898 (27.1)
Blood & blood-forming organs	380 (11.5)
Respiratory system	222 (6.7)
Musculoskeletal system	147 (4.4)
Nervous system	146 (4.4)
Genitourinary system	101 (3.1)
Sensory organs	63 (1.9)
Dermatological	51 (1.5)
Systemic hormones	32 (1.0)
Antineoplastics	30 (0.9)
Anti-infective and various	18 (0.5)
Diagnosis (based on ICD-11 classification)	**Number of diseases (%)** (***n*** = **1845)**
Circulatory system diseases	826 (44.8)
Endocrine, nutritional and metabolic diseases	286 (15.5)
Genitourinary system diseases	182 (9.9)
Musculoskeletal system and connective tissue diseases	105 (5.7)
Respiratory system diseases	97 (5.3)
Gastroenterology diseases	69 (3.7)
Other diseases	280 (15.2)

Factors associated with high MRCI were tested using univariate analyses. Subsequently, a multivariate linear regression model was applied to adjusting for covariates. As shown in Table 3, the multivariate linear regression model exhibited that CCI score and the presence of polypharmacy were the significant determinants of high MRCI score.

**Table 3:** Factors associated with high medication regimen complexity score using multivariate linear regression

Independent variables	β	95% Confidence Interval	*p* value
**Charlson Comorbidity Index**	**0.500**	**0.239–0.760**	**< 0.001**
Age	-0.032	-0.135–0.072	0.549
Gender			
Male	0.661	-0.341–1.662	0.195
Female	1	(Ref)	(Ref)
Polypharmacy			
No polypharmacy (3–4 medications)	1	(Ref)	(Ref)
**Polypharmacy (5–9 medications)**	**5.772**	**4.073–7.471**	**< 0.001**
**Hyperpolypharmacy (≥ 10 medications)**	**14.929**	**12.971–16.888**	**< 0.001**
Marital status			
Married	-0.747	-2.105–0.610	0.262
Other (widowed, divorced & single)	1	(Ref)	(Ref)

*R*^2^ = 0.492, Adjusted *R*^2^ = 0.482

### Factors associated with medication nonadherence and treatment satisfaction

**Fig. 2:**
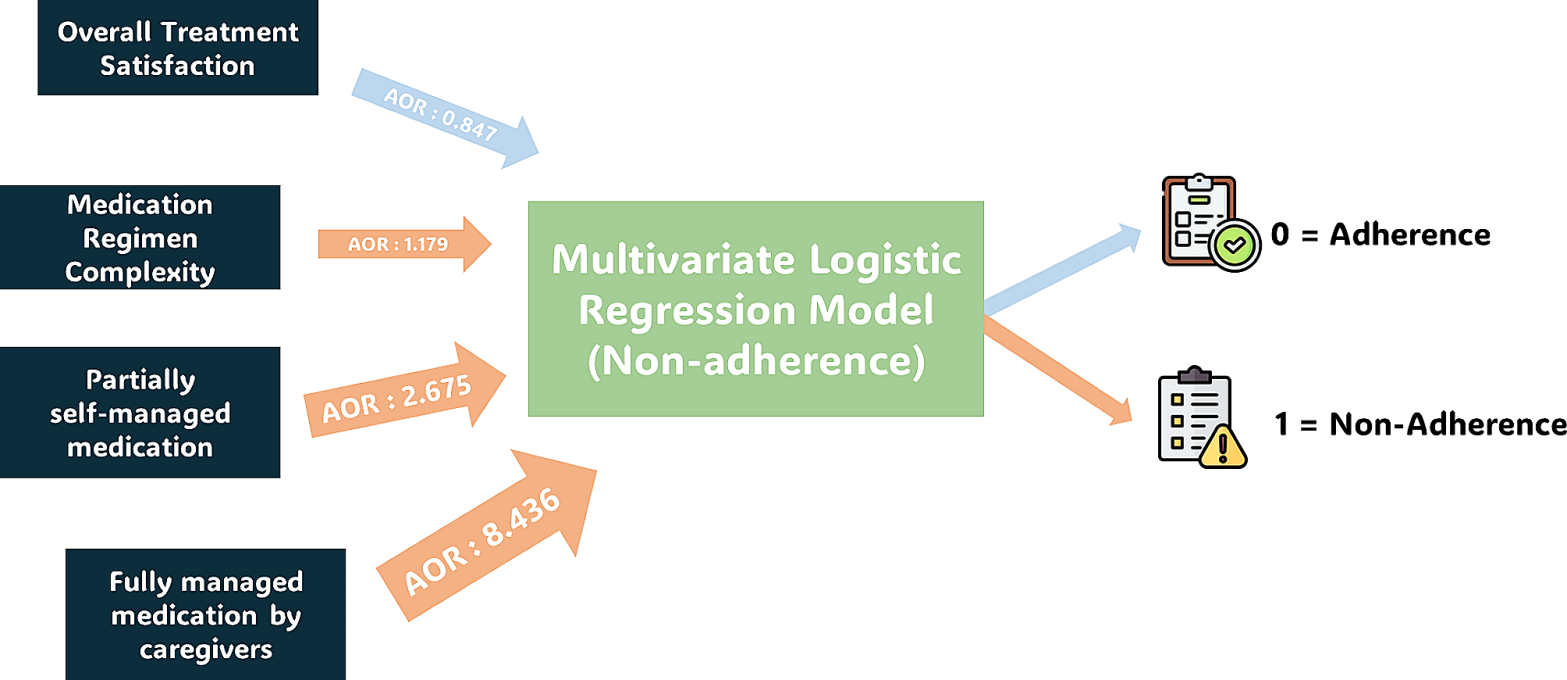
The significant determinants of nonadherence using multivariate logistic regression. The larger the arrow is the greater the impact is. AOR: adjusted odd ratio

Table 1 depicts the results of the chi-square test and t-test of medication adherence as the dependent variable with the involvement of several independent variables. Various factors were significantly associated with medication nonadherence. After controlling for covariables using multivariate logistic regression analysis, four factors significantly predicted nonadherence to the medications:MRC (AOR = 1.179, 95% CI: 1.064–1.306, *p* = 0.002), overall treatment satisfaction (AOR = 0.847, 95% CI: 0.811–0.884, *p* < 0.001), partially self-managed medication (AOR = 2.675, 95% CI: 1.259–5.685, *p* = 0.011) and fully managed by family members/caregivers (AOR = 8.436, 95% CI: 2.003–35.524, *p* = 0.004). All assumptions needed to apply the binominal logistic regression were met before running the model. The binominal logistic regression model statistically and significantly predicted patient nonadherence. Additionally, the area under the ROC curve of 96.0% and *p-*value of 0.489 for the Hosmer and Lemeshow test indicates that this logistic regression model is a good fit for the data. Figure 2; Table 4.

**Table 4:** Variables included in the nonadherence logistic regression model

Independent variables	Adjusted Odds Ratio (AOR)	95% Confidence Interval (CI)	*p* value
**Medication Regimen Complexity**	**1.179**	**1.064–1.306**	0.002*
**Treatment Satisfaction (Overall)**	**0.847**	**0.811–0.884**	< 0.001*
Charlson Comorbidity Index	0.945	0.773–1.115	0.580
Medication management			
Self-managed	1	(Ref)	(Ref)
**Partially self-managed**	**2.675**	**1.259–5.685**	0.011*
**Fully managed by family members/caregivers**	**8.436**	**2.003–35.524**	0.004*
Number of medications per patient/polypharmacy	1.120	0.882–1.421	0.354
Other variables			
Age	1.026	0.946–1.113	0.531
Gender			
Male	1	(Ref)	(Ref)
Female	0.992	0.450–2.189	0.985
Marital status			
Married	1	(Ref)	(Ref)
Other (widowed, divorced & single)	1.046	0.390–2.808	0.929
Geographical location			
Urban	1	(Ref)	(Ref)
Rural	0.889	0.407–1.943	0.768
Education level			
College/university	1	(Ref)	(Ref)
Secondary school	1.085	0.326 – 3.612	0.894
Primary school & other (no formal education)	2.794	0.610 – 12.282	0.186
Income status			
< RM 1000	1.242	0.699 – 2.209	0.394
RM 1000 - RM 2500	0.357	0.421 – 1.476	0.195
RM 2501 - RM 4000	1.444	0.431 – 4.387	0.551
RM 4001 - RM 4999	0.602	0.164 – 2.208	0.444
≥ RM 5000	1	(Ref)	(Ref)

Omnibus Test of Model Coefficient (Chi-square = 372.85, *p* < 0.001)

Hosmer and Lemeshow Test [χ^2^(8) = 7.453, *p =* 0.489]

Area under Receiver Operating Characteristic (ROC) curve = 0.960 (95% CI: 0.944,0.976)

Table 5 shows univariate and multivariate analyses that were conducted to determine the significant predictors of overall treatment satisfaction. After adjusting for other covariates, only three predictors were significantly associated with overall treatment satisfaction, namely MRC (β = -1.395, 95% CI: -1.635 – -1.155, *p* < 0.001), CCI (β = -0.746, 95% CI: -1.303 – -0.189, *p =* 0.009) and self-managed medication (β = 5.554, 95% CI: 1.634–9.474, *p* = 0.006). Conclusively, the value of *R*^2^ in this multivariate linear regression model is 50.3%, and the combination of the medication regimen complexity, self-managed medication and CCI variables could explain the variance in overall treatment satisfaction in this model.

**Table 5:** Univariate and multivariate linear regression to identify predictors of treatment satisfaction

Independent variable	Univariate analysis				Multivariate analysis		
	β	95% CI	*p* value		β	95% CI	*p* value
**Medication Regimen Complexity**	**-1.462**	**-1.612, -1.311**	**< 0.001**		**-1.395**	**-1.635, -1.155**	< 0.001*
Number of medications per patient/polypharmacy	-2.979	-3.462, -2.497	< 0.001		0.577	-0.075, 1.230	0.083
**Charlson Comorbidity Index**	**-2.534**	**-3.189, -1.879**	**< 0.001**		**-0.746**	**-1.303, -0.189**	0.009*
Medication management							
**Self-managed**	**17.383**	**13.019, 21.747**	**< 0.001**		**5.554**	**1.634, 9.474**	**0.006**
Partially self-managed	3.905	-0.565, 8.375	0.087		1.323	-2.351, 4.997	0.479
Fully managed by family members/caregivers	(Ref)				(Ref)		
Marital status							
Married	3.983	0.464, 7.502	0.027		2.283	-0.299, 4.864	0.083
Other (widowed, divorced & single)	(Ref)				(Ref)		
Geographical location							
Urban	0.146	-2.870, 3.071	0.922		1.334	-0.780, 3.447	0.216
Rural	(Ref)				(Ref)		

*R*^2^ = 0.503, Adjusted *R*^2^ = 0.494

95% CI = 95% Confidence Interval for β

### The impact of MRC on medication adherence through treatment satisfaction

The mediation analysis indicates a significant effect of the indirect pathway of medication regimen complexity on nonadherence through treatment satisfaction (β = 0.230, 95% CI = 0.175–0.316). The direct pathway demonstrates a significant effect of medication regimen complexity on nonadherence (β = 0.211). Since both pathways showed significant effects, treatment satisfaction partially mediated the effect of medication regimen complexity on nonadherence.

## Discussion

This study shows that 51.0% of the older adult patients visiting outpatient clinics were nonadherent to their chronic medications. Similar findings reported from Portugal, Singapore and the USA revealed that the prevalence of nonadherence among older adults was 56.3%, 60.0% and 54.8%, respectively [28–30]. Additionally, a recent systematic review and meta-analysis demonstrated that the pooled prevalence of nonadherence among older adult patients in Malaysia is 60.6% [4]. Hence, all studies reported a comparable prevalence to ours, with more than half of the older adult patients being nonadherent.

One meta-analysis study indicated that the prevalence of polypharmacy among the Malaysian older adult population ranged from 20.3 to 100% [4]. Myriad definitions of polypharmacy terminology have been implemented in various studies, which signify the wide range of polypharmacy prevalence reported in this meta-analysis study. Their study also demonstrates that the pooled prevalence of polypharmacy from multiple studies is 49.5%, which means that nearly half of Malaysian older adult patients are polypharmacy [4]. Nevertheless, the prevalence of polypharmacy in the current study was found to be 90.2%, which means that the majority of patients visiting our study site are exposed to polypharmacy. This difference could be attributed to the fact that the SASMEC@IIUM is a teaching hospital and tertiary care setting in which most of the participants in this population are referred to various outpatient specialist clinics. The pooled prevalence (49.5%) reported in the above-mentioned meta-analysis was derived from heterogeneous studies conducted in primary, secondary and tertiary healthcare settings, which may affect the number of prescribed medications per patient. Moreover, the reported CCI score in our study was much higher than that reported in the other studies. High number of comorbidities is the basis for the increase in polypharmacy prevalence as most of our patients diagnosed with high number of comorbidities (high CCI score). A study conducted among older patients with chronic pain in Germany found a significant association between CCI score and polypharmacy [31].

Next, our study showed an average CCI score of 5.4, indicating a high and severe comorbidity burden within our study population. Another study conducted in a tertiary care hospital in Thailand among the geriatric population reported a score of 4.7, which implies that our study has a slightly higher comorbidity burden [32]. It is common for older adult patients to be diagnosed with multiple morbidities and subsequently have a high CCI [33].

The reported mean MRCI score in this study was 17.38 (SD = 7.07), which can be categorized into medium complexity (15.5–20) [20]. In comparison, two studies that evaluated MRC among Malaysian older adults diagnosed with acute infections (i.e., UTI and RTI) reported low complexity (≤ 15.0), which were 14.0 and 11.8, respectively [34, 35]. Other studies also evaluated MRC among chronic kidney disease (CKD) older patients in Australia and Norway, with reported higher MRCI scores of 27.0 and 22.8, respectively, which are classified as high complexity (≥ 20.5) [36, 37]. A wide range of medication regimen complexity scores reported in distinct studies explained the variation in medication regimens across various patient populations and healthcare settings. Other than that, our study was also able to identify the significant determinants of high MRCI score using a regression model, which are the CCI and the presence of polypharmacy. A possible explanation for this might be that most comorbid older adult patients in this present study were severely morbid and diagnosed with various diseases, leading to more prescribed concurrent medications according to their management plan. Hence, the complexity of medication regimens arises as a result of this phenomenon. This finding is broadly supported and consistent with other literature, in which they also reported that increasing CCI scores and polypharmacy were associated with higher MRCI scores [36, 38].

Furthermore, our study reported a mean of 73.91 (SD = 15.23) for overall treatment satisfaction, which is comparable to several similar studies evaluating treatment satisfaction using TSQM outside Malaysia, such as Italy, Lebanon, and South Korea [39–41]. The average overall treatment satisfaction ranged from 59.28 to 77.36, with the highest satisfaction score reported by Sakr et al. (2018), who conducted the study among Lebanese general elderly population [41]. Meanwhile, a study by Byun et al. (2019) among postmenopausal osteoporosis in South Korea reported the lowest satisfaction score [40]. The varied satisfaction score might be due to the type of population selected in the sample size (i.e., general population Vs diseases-specific population). Thus, our treatment satisfaction score falls within the range of similar studies, indicating that our study population is reasonably satisfied with the treatment, which can be considered a positive benchmark of the treatment effectiveness provided to the patients.

Our research proved that increasing MRC is a significant predictor of nonadherence. In comparison, several reports have examined the negative association between MRC and medication adherence among older patients across groups of diseases and localities, in which the majority of the studies indicate that MRC is negatively associated with medication adherence [20, 28, 42–45]. Meanwhile, a few studies found no negative association between these variables [30, 36, 46]. This finding further describes that complex medication regimens could increase the cognitive load among older adult patients, with some of them having difficulty comprehending the whole medication instructions due to cognitive deterioration. Therefore, it also leads to regimen confusion between the medications taken and results in nonadherence among the older adult population.

The current study demonstrates that treatment satisfaction is a significant predictor of medication adherence after adjusting for other covariates. This finding is consistent with several studies that show a positive association between these variables among older adult patients [47–50]. Hence, this may explain why patients who are satisfied with their medication have enhanced motivation due to their expectations being fulfilled. Therefore, good treatment satisfaction suffices as a positive reinforcement for patient adherence.

Additionally, this study indicates that MRC is a significant predictor of treatment satisfaction even after controlling for other variables. In fact, a limited number of studies assess this association especially in older adults. A possible explanation for this might be that MRC could relate to the inconvenience of taking medication. Older adult patients often experience such difficulty while taking their medication, especially with complex instructions for certain medications. Therefore, the patient may suffer from frustration and demotivation, which could lead to low treatment satisfaction. Our result aligns with a study conducted on patients with hepatitis C. The researchers found that reducing regimen complexity was associated with increasing in patients’ satisfaction with the treatment [51]. However, a simple reduction of dosing frequency from twice to once daily for one medication did not significantly improve treatment satisfaction in patients with heart failure [52].

The mediation analysis revealed that treatment satisfaction partially mediated the association between MRC and nonadherence. Treatment satisfaction acts as a partial mediator, which means that the presence of this factor accounts for the partial influence of MRC on non-adherence. There is only part of the association between MRC and non-adherence that can be explained by the patient’s satisfaction with their treatment via indirect effect in mediation analysis. In other words, a more complex medication regimen leads to lower treatment satisfaction and, subsequently, poorer medication adherence. Moreover, the direct effect of MRC to non-adherence in mediation analysis is also significant, which means that the association may not involve the mediation of treatment satisfaction. This means that in addition to its direct effect on medication adherence, MRC also indirectly affects medication adherence through patient’s satisfaction on the treatment. This finding is consistent with the studies by other researchers, which suggest that nonadherence is affected by MRC and treatment satisfaction [9, 10]. However, neither study could explain the mediating role of treatment satisfaction in this association due to the scarcity of studies assessing this association between these three variables altogether. Thus, this finding supports our hypothesis in the proposed framework, in which our study expected the role of treatment satisfaction as a mediating variable in this association. Future research should focus on determining treatment satisfaction as a mediator of MRC in medication adherence. Emphasizing the evaluation of treatment satisfaction could promote involving the patient in the routine clinical decision. Therefore, the patient’s preferences will be prioritized, and medication regimen can be personalized to make positive impact on medication adherence among older adult patients.

Both regression models indicate that medication management is significantly associated with medication adherence and treatment satisfaction. Participants who were either partially or fully dependent on other people – in terms of management medications – had a significantly higher possibility of being nonadherent to their medications compared with independent patients. There are similarities between our findings and those described by other researchers [53]. A possible explanation for this result is that older patients who managed medications by themselves have a higher level of engagement and control over their medications. However, those who rely on the caregiver cannot fully engage with their treatment plan and lose control over their medications, which leads to nonadherence. Additionally, communication barriers between patients and caregivers might contribute to misunderstanding and lack of clarity regarding the treatment, which could also influence adherence [54]. It is worth noting here that the assessment of patients’ dependence on medication management was based only on one question. Although the question was clear and straightforward, it may not reflect the actual daily behavior of the patients. Therefore, further studies should implement a valid measurement tool for determining the type of medication management.

According to Ulley et al., another covariate potentially associated with nonadherence and treatment satisfaction among older adults is the number of medications per patient/polypharmacy [55]. Initially, our findings showed that polypharmacy is associated with nonadherence and treatment satisfaction using univariate analysis. However, multivariate analyses demonstrated that polypharmacy is not a significant predictor of nonadherence or treatment satisfaction after controlling for other variables. The basis behind the lack of association might be due to the high prevalence of polypharmacy (> 90%) in the study population, making it an indiscriminating factor between adherent and nonadherent patients. Another reason could be the presence of other strong predictors in the models, which influence nonadherence and treatment satisfaction in this population.

In addition, our incidental findings showed that CCI is a significant predictor of treatment satisfaction, which might be because severely morbid patients have poor prognosis for specific types of diseases (e.g., cancer, T2DM complications), leading to low treatment satisfaction. A study conducted in China supported our finding, in which they reported that the severity of the disease contributes to lower medication satisfaction among COPD patients. Their finding demonstrated that those with more severe conditions tend to have lower satisfaction due to the high occurrence of symptoms [56]. Consistent with our finding, a study conducted among diabetic patients in Ethiopia also signifies that complications and multimorbidity were the key determinants of lower degree of treatment satisfaction [57]. Thus, further studies should focus on assessing this association to identify the actual basis between these variables among older adult patients.

This present research can identify several limitations throughout the whole study process. First, the information on the additional instructions about the medications prescribed is not extensively updated in the electronic health record system; thus, MRCI could be underestimated. In addition, our study only considers the subjective measure of medication adherence using the self-reported measurement tool, in which patients tend to overestimate the level of adherence. Next, our research was conducted in a unicentric setting in Kuantan, Pahang, limiting the generalization of the findings to the Malaysian older adult population. In addition, even though convenience sampling is quick, cost-effective and ease of access way for sampling method, the main limitation is the potential for sampling bias. Thus, based on setting and convenience sampling, the generalizability of study is limited. A future multicenter longitudinal study implementing objective assessment for nonadherence is recommended.

## Conclusion

The study explored the association between medication regimen complexity, treatment satisfaction and medication adherence among older adult patients in Pahang, Malaysia. Approximately half of the population was not adherent to their medications and had medium complexity regimens. The notable findings in this study showed that MRC, overall treatment satisfaction, and partial and full dependence on others to manage medications are significant predictors of medication nonadherence. Furthermore, the significant determinants influencing overall treatment satisfaction were MRC, CCI and self-managed medications. Last, treatment satisfaction partially mediated the association between MRC and nonadherence.

This study suggests that simplifying the medication regimen and involving patients in the treatment plan could be part of the strategy to solve the nonadherence issue in older adults. Future interventional studies are warranted to prove the above assumption. Other than that, recognizing patient-reported outcomes such as treatment satisfaction could emphasize the importance of tailoring the medication regimen according to the patient’s experiences. Therefore, ensuring the patient is satisfied with their treatment may address the issue of nonadherence among the older adult population.

## Data Availability

No datasets were generated or analysed during the current study.
